# Foray into Concepts of Design and Evaluation of Microemulsions as a Modern Approach for Topical Applications in Acne Pathology

**DOI:** 10.3390/nano10112292

**Published:** 2020-11-19

**Authors:** Marina-Theodora Talianu, Cristina-Elena Dinu-Pîrvu, Mihaela Violeta Ghica, Valentina Anuţa, Viorel Jinga, Lăcrămioara Popa

**Affiliations:** 1 Department of Physical and Colloidal Chemistry, Faculty of Pharmacy, “Carol Davila” University of Medicine and Pharmacy, 020950 Bucharest, Romania; marina-theodora.talianu@drd.umfcd.ro (M.-T.T.); cristina.dinu@umfcd.ro (C.-E.D.-P.); valentina.anuta@umfcd.ro (V.A.); lacramioara.popa@umfcd.ro (L.P.); 2 Department of Clinical Sciences, no.3, Faculty of Medicine, “Carol Davila” University of Medicine and Pharmacy, 020021 Bucharest, Romania; viorel.jinga@umfcd.ro

**Keywords:** dermatologic diseases, acne therapy, skin target, modern systems, microemulsions, microstructure, API delivering, emulsifiers, penetration enhancers, evaluation methods

## Abstract

With a fascinating complexity, governed by multiple physiological processes, the skin is considered a mantle with protective functions which during lifetime are frequently impaired, triggering dermatologic disorders. As one of the most prevalent dermatologic conditions worldwide, characterized by a complex pathogenesis and a high recurrence, acne can affect the patient’s quality of life. Smart topical vehicles represent a good option in the treatment of a versatile skin condition. By surpassing the stratum corneum known for diffusional resistance, a superior topical bioavailability can be obtained at the affected place. In this direction, the literature study presents microemulsions as a part of a condensed group of modern formulations. Microemulsions are appreciated for their superior profile in matters of drug delivery, especially for challenging substances with hydrophilic or lipophilic structures. Formulated as transparent and thermodynamically stable systems, using simplified methods of preparation, microemulsions have a simple and clear appearance. Their unique structures can be explained as a function of the formulation parameters which were found to be the mainstay of a targeted therapy.

## 1. Introduction

Observed as a sophisticated mantle, the skin is designed as a first line interface that binds our body with the external environment [1]. Its coordinative character for vital functions is associated with fragility and sensitivity [2,3]. Each of these features reflect our evolution from new born existence until elderly stage, being extremely well defined in the pathological state, when the unveiled properties of the skin and its internal mechanisms will need special attention courtesy of patient and his doctor [4,5]. Dermatologic diseases are widespread over the world and have a high impact on the life quality of the patient with both physical and psychosocial repercussions [6]. The most encountered affections with a high prevalence are: eczema, acne, psoriasis, fungal or viral skin affections, autoimmune conditions, ulcerative conditions, skin burns, skin traumas, pigmentary disorders, keratinocyte carcinoma or malignant skin melanoma pathologies [7,8]. In agreement with the Global Burden of Disease Project [9], skin diseases occupied the fourth place in 2010 and 2013 as non-fatal skin injuries. These are determined by genetic factors, pre-existent systemic diseases, geographical area and socioeconomic influences such as the impairment of life quality because of the lack of access to medical care [9,10].

**Figure 1:**
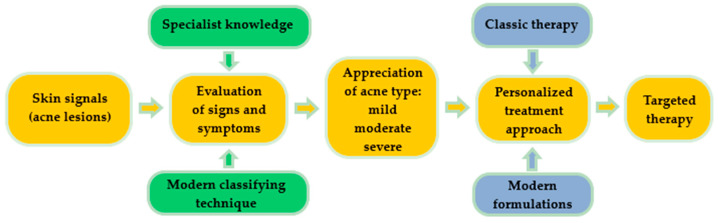
Schematic representation concerning the impact of a right diagnosis for a personalized treatment in a dermatologic disease.

Acne is a common chronic skin condition and has been reported as one of the 10 most prevalent diseases worldwide. Its recurrent character is frequently encountered in clinical cases [11]. A proper treatment will always be suggested after a right and conclusive diagnosis, as can be observed in Figure 1. Classically, skin signal is observed in a visual inspection of the specialist. In order to increase the quality of acne diagnosis, modern classifying techniques based on convolutional neural networks are focused on the imaging and differentiation of acne lesions from healthy tissue [12]. By recognizing a skin pattern and appreciating acne evolution, personalized treatments can be created based on classical or modern pharmaceutical formulations, gently chosen to assure a targeted therapy.

Numerous therapeutic approaches were initiated to enhance the bioavailability of dermatologic drugs at skin site in the hope to overcome the barrier effect of stratum corneum. The avoidance of oral administration of systemic drugs is also pursued, as well as attempts to integrate them in topical formulations [13].

Here, nanotechnology can put its signature, defining new ways to design proper formulations with superior therapeutic outcomes [14]. In the field of nanocolloids, microemulsions are appreciated as systems that can satisfy a high number of exigencies in matter of drug delivery—ease of preparation using a small number of ingredients like oil, water, a surfactant and a cosurfactant [15]; integration on this way of hydrophilic or lipophilic actives that can be solubilized in one of the phases [16]; generation of clear and stable dispersions with nanometric particles that can pass through biological membranes [15]—are considered a suitable platform for nanoparticle synthesis [17]. Furthermore, the use of biocompatible sources of oils like vegetable oils [18], natural surfactants [19] or biopolymers [20] can place microemulsions in the category of green alternatives in topical or systemic use of drugs with targeted action.

Early contributions in 1990s were focused on the projection of oral microemulsionate systems as vehicles based on medium chain fatty acids and their salts, able to promote calcein absorption [21]. For testosterone propionate, ample solubilization screenings were performed to find the appropriate oil phase. It was concluded that oil-based solubilizer agents are preparation key factors that can affect the internal behaviour of microemulsions and must be judiciously studied and selected for preparation [22].

Multiple applications in biomedical field were hypothesized and studied considering as effective the delivery of microemulsions in the human body. Several discoveries with positive results can be mentioned: in ophthalmology [23], for ocular delivery of retinol [24] or sacha inchi oil for dry eye treatment [25]; intranasal delivery for zidovudine [26]; sublingual delivery of insulin [27]; application of fusidic acid in wound healing [28]; vaginal delivery for fluconazole [29]; transdermal drug delivery [30]; cosmetics [31]; self-microemulsifyied systems with in situ generation of microemulsion in biological fluids for oral delivery [32]. In topical therapy of dermatologic conditions, an increased solubility and bioavailability was obtained for molecules like: cyclosporine in psoriasis [33]; ceramides for skin restructuration [34]; imiquimod in actinic keratosis or basal cell carcinoma [35]; penciclovir [36], acyclovir for herpes virus simplex cutaneous infection [37]; and tenoxicam for arthritis alleviation [38].

Designing microemulsion systems seems also to be an ideal approach for anti-acne drug targeting and must be taken under attention with respect to skin structure peculiarities and the current status of topical treatments, offering a background concerning the evolution of topical formulations and the impact of microemulsions on anti-acne drug delivery. The effect of microemulsions at skin sites can be corroborated with their structural profile. The influence of composition type, the method of preparation, the presence of penetration enhancers or thickening agents represent the main criteria followed by microemulsion design and further described in the present review [39].

## 2. Skin Structure and Implications in Topical Delivery of Active Molecules

The human body keeps its complexity surrounded by an organ with a surface of approximately 2 m^2^, defined as a natural barrier. The skin is imagined as a frontline interface between the body and the external medium [40,41].

The main functions of the skin are considered as follows: it assures an equilibrium between the intake and the loss of water and electrolytes with an important role in hydration mechanisms, it regulates the body temperature, and it gives protection against ultraviolet (UV) radiation with the help of stratum corneum [41,42]. Furthermore, the immune and bacteriostatic mechanisms at skin site are implied in a defense against microorganisms, combating skin infections [40,43].

**Figure 2:**
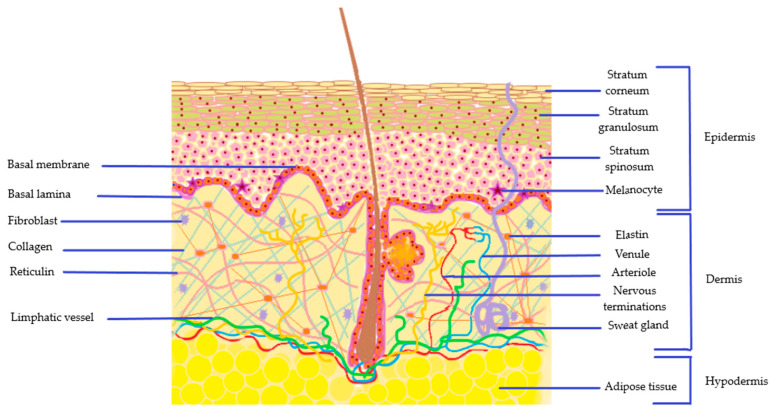
Anatomical structure of skin with the most important components of epidermis, dermis and hypodermis layers.

The architecture of the skin presented in Figure 2, is based on three types of layer with a specific cellular composition that promotes protection: the epidermis, dermis, and hypodermis [43,44].

### 2.1. The Epidermis

The outermost layer of the skin, the epidermis is composed from a multi-layered cornified epithelium, with five types of strata, disposed in a specific manner. These are classified from the inside part through the outer as basal lamina with stratum germinativum, stratum spinosum, stratum granulosum, stratum lucidum and stratum corneum [45]. The basal membrane, with its unicellular structure with cubic keratinocyte cells that assure a permanent regeneration of the skin, is localized near dermis zone and is continued with a polyhedral cells’ arrangement of stratum spinosum in 8–10 layers. Both strata are enriched in specialized cells which are implied in skin deposition of melanic pigments. In addition, Merkel cells have sensorial properties, while Langerhans cells are implied in immune mechanisms. Stratum spinosum is continued with stratum granulosum characterized by 6 layers with elongated and nucleated cells which contain keratohyalin complexes and lamellar bodies [46,47]. Thus, keratohyalin has a key role in the development of the cornified envelope due to the presence of three main proteins profilaggrin, loricrin and involucrin which are implied in biochemical reactions that sustain the epidermal evolution and integrity of the external layer, the stratum corneum [48].

The stratum lucidum is found as a translucent layer and is well defined on palms and soles with multiple strata based on flattened keratinocytes [48].

The stratum corneum (SC), the natural barrier of the body has a thin dimension of 10–20 μm. The thin appearance is assured by a balanced and permanent cellular turnover. Its structure is based on the presence of corneocytes filled with keratin (the main structural protein), water molecules and a natural moisturizing factor which is an essential complex for SC functions. The corneocytes known also as death cells are embedded in dense lipidic regions based on ceramides, cholesterol and fatty acids, which will promote diffusional resistance [44,49]. It is important to note that the generation of a lipidic matrix is based on multiple self-assembly processes which begin from polar compounds. Here are distinguished sphingomyelin, glucosylceramide, cholesterol, and phospholipids [50,51]. The interconnections between corneocytes are made by corneodesmosomes, structures derived from desmosomes units as a result of differentiation process in the upper layer of the stratum granulosum [52]. The normal function of corneodesmosomes can be compromised in skin dermatoses ending with an additional accumulation of stratum corneum or a loss in its consistency. Corneodesmosomes will not be normally degraded by an enzymatic route anymore and will persist in the upper layers generating hyperkeratosis or xerosis [53]. The modified state of the skin is highly influenced and correlated with the presence of natural moisturizing factor in corneocytes and more profoundly with the activity of profilaggrin and filaggrin proteins of keratohyalin domain.

To conclude, moisturizing formulations are suitable to use as adjuvants beyond therapeutic agents to mitigate the imbalances that affect the normal function of epidermis [54,55]. To exemplify, in a study of Lynde et al., it was suggested that ceramide-based moisturizing systems can contribute to the recovery of skin balance for acne patients that have experienced disorders like dry skin, xerosis and irritation or skin barrier dysfunctions following a topical or a systemic anti-acne treatment [56].

### 2.2. The Dermis

Localized in the middle area of the skin structure, with a papillary and a reticulate domain, the dermis has a major function in epidermis nutrition. The basal membrane of epidermis assures a functional interdependence between both layers. Fibroblasts are the main cells of dermis level beyond mast cells, histiocytes and adipocytes [48,57]. Fibroblasts assure connections with the epidermal layer, being recognized basically as specialized cells that confer skin tightness and elasticity [58].

Beyond these important cellular interconnections that assure a normal function at skin level, the connective tissue found in dermis level is composed of collagen, elastin and reticuline fibers. These structural proteins are arranged in networks that include an internal matrix based on glycosaminoglycans, proteoglycans and glycoprotein molecules which ensure a balanced hydration level [59]. A key molecule with hydration and regeneration properties implied in skin metabolism is hyaluronic acid [60]. Overall, the entire area of dermis provides structural integrity, elasticity and mechanical support, with a contribution to skin barrier protection against external harmful factors [61].

As a vascularized domain, at dermis level are made connections with the internal environment through the system of venules and arterioles. On the same side is found the lymphatic flow and a complex frame with nervous terminations that assure sensorial activity. Here, the tactile, thermic and pain sensitivities are part of fundamental structures of the body that help humans to adapt, react and protect against a live medium. The regeneration processes at skin site are highly influenced by the activity of peripheral nervous system and the presence of neuromediators that interact with cellular structures [61,62].

The complexity of the dermis is completed by the presence of pilosebaceous units and sweat glands. These structures are implied in sebum secretion and sweating with a pathological pattern specific to dermatologic conditions like acne, alopecia, or hyperhidrosis [63,64].

### 2.3. The Hypodermis

The hypodermis, known also as a subcutaneous layer, is mainly composed of loose connective tissue and adipose tissue with functions that assure mechanical support, protection against shocks, thermoregulation, and energy storage properties. At this site is localized the appendageal origin, as a continuation of dermis functional structures. The adipose tissue, which comprises around 75% of the total amount of substance is sustained by the presence of collagen. Its composition is completed with 20% water and 5% proteins [44,65].

### 2.4. Topical Therapy as a Route of Administration

A variety of administration routes are accessed in dermatologic therapy: topical treatment with administration on skin, systemic treatment which comprises the oral administration, parenteral administration and transdermal route access; local administration using injections in skin layers, in association with interventions based on chemical and physical methods [66,67]; intralesional injections [68].

Topical therapy is appreciated as a route of administration, mostly preferred by patients due to its easy-to-use character [69]. This route is non-invasive, the patient will apply the treatment at home without the intervention of a medical professional, obtaining benefits in what concerns the adherence. In addition, the systemic absorption is limited and the first pass effect specific to oral delivery is avoided. On the other hand, the limitations of skin path are related to the characteristics of actives. As an example, the bioavailability of high molecular weight substances with low lipid solubility will not have a superior value. Thus, skin retention will be poorer. Another limitation implies a possible passage of a substance through the skin with lesions when the natural barrier is compromised, with an increase of side-effect events [70,71]. It is important to appreciate that the active substances, their concentration, the excipients selected in the formulation of the vehicle, the type of skin condition, the type of lesion, and the possible incompatibilities are factors that must be taken into account when a therapeutic effect is expected [72]. As a function of the pharmaceutical formulation, the conventional topical systems used in dermatologic therapy are: liquid dosage forms including solutions, suspensions and emulsions which can be oil in water (O/W), water in oil (W/O) or multiple types; moreover, semi-solid formulations are distinguished as creams, pastes, ointments and gels [72,73].

The evolution of topical therapy systems must be considered an essential process which encompasses the knowledge of both the advantages and limitations of classical formulations being in correlation with the biopharmaceutical relevance of an active pharmaceutical ingredient (API) [74]. Regarding the properties of a substance in matter of solubility and permeability across the membranes of the body—in our case, the skin with its complexity—the delivery in a specific stratum is dependent on the type of the selected vehicle [75]. The physicochemical characteristics of each component and their interactions are important to be noted in a formulation process and analyzed in correlation with the anatomical site of action [76,77].

Thus, the main barrier in the mechanism of diffusion at skin level is the SC due to its organization based on flattened corneocytes units surrounded by a double-layer lipid stratum with a high level of ceramides, cholesterol and fatty acids [78,79]. Even if the SC is characterized like a thin layer with a dimension of 10–20 μm, the concentration of diffused active substances from a classical formulation has often a low value when the vehicle is not suitable for delivering the actives to the affected site [80]. An ideal substance that can pass the natural barrier may have a balanced pattern based on the existence of both lipophilic and hydrophilic properties, with a good solubility, a low melting point value and a small molecular weight (<500 Da) [75,81,82].

**Figure 3:**
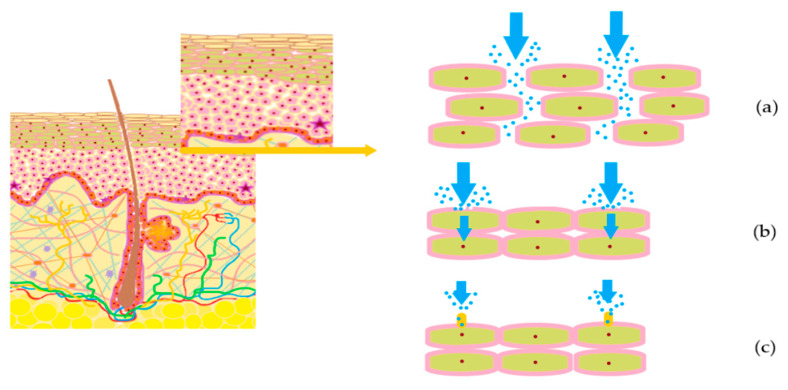
Pathways of particles passage for hydrophilic substances: (**a**) intercellular passage of particles between epidermal cells; (**b**) transcellular passage of particles using lipid defects in epidermal cells; (**c**) transcellular passage of particles through micropores of epidermal cells.

In the penetration process were described four important pathways that can be handled in the case of hydrophilic (polar) or lipophilic (non-polar) substances. Firstly, the intercellular passage is observed as a route between the cells which can explain the water hydration property. It was observed that hydrophilic permeants can pass into the lower skin layers via lipid defects on a transcellular pathway, or through micropores in the appendageal walls across a polar pores’ pathway. [78,83]. These three pathways are presented schematically in Figure 3.

For lipophilic substances the passage through the pilosebaceous unit can be reached when there are associated percutaneous penetration enhancers, capable of creating pores by a temporary destruction in lipid stratum [78,83]. Recent findings suggest that transfollicular pathway could be a solution for the delivery and accumulation of actives with action in acne or alopecia, comprising localized effects [84,85].

**Figure 4:**
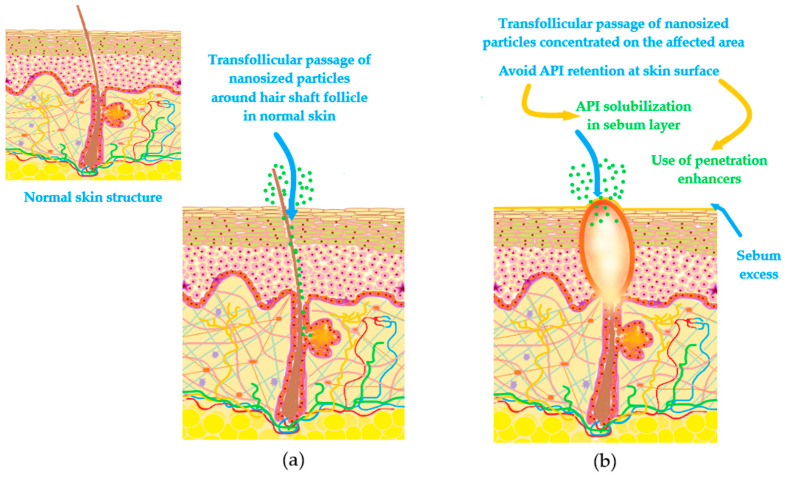
Transfollicular passage of nanosized particles in skin structure: (**a**) transfollicular passage around hair shaft follicle in normal skin; (**b**) modified passage accessing hair shaft follicle in acne affected skin with comedonal development and inflammation flare.

In acne-prone skin, the delivery process of APIs must be concentrated at follicular level, recognized as a central structure of acne development. The challenge in the ideal delivery consists in the fact that normal skin structure is impaired by processes like the increase of transepidermal water loss, coupled with an increase of sebum secretion [86]. Moreover, follicle blockage is considered another obstacle that will harden the passage of drugs, decreasing their bioavailability [86,87,88]. Quantifying these observations, for an impaired skin it is harder to obtain a therapeutic effect in a targeted place. In reverse, skin will be more predisposed to many adverse reactions encountered in clinical practice like erythema, xerosis or dermatosis [87]. In this way, some directions were proposed to facilitate the passage of APIs at follicle level. Firstly, solubilization of drug in sebum secretion may be possible only for lipophilic drugs. Secondly, a better alternative for both lipophilic and hydrophilic drugs is the selection in the formulation process of vehicles capable to alter sebum structure without inducing skin damage. Amphiphiles and alcohols, extensively studied as penetration enhancers, are good candidates for this purpose [88]. A superior delivery at follicle level, with lower adverse events was observed for retinoids prepared as vesicular nanosystems, nanoparticles, nanoemulsions or microemulsions, compared with conventional formulations [88,89]. Similar observations were found for benzoyl peroxide prepared in polymeric micelles versus conventional gel [90]. Figure 4 shows cellular passages for drug molecules in a nanoparticulate form, with emphasis on transfollicular path accessed by nanoparticles in normal skin, compared with acne-prone skin frequently characterized by pore blockage, comedonal development and inflammatory processes.

## 3. Current Topical Formulations in Acne Therapy

### 3.1. Short Characterization and Prevalence among Population

Acne is defined as a chronic inflammatory disease, generally dispersed on face, neck, back and rarely on chest area, with differences in the development among affected patients [91]. The most encountered form of acne in clinical practice is acne vulgaris [92]. Acne pathology has a prevalence of 85% in adolescent age, being extended also in the adulthood [93]. Adult females and males over 25 years old experiencing this type of skin disease were affected in the teenage period. An early development of acne in adolescent age was followed by a recurrence or exacerbation in adult period [94]. As a short statistical information from a university-based survey of Collier et al. made on 1013 subjects of 20 years and older (mean age 48.0 ± 16.7), 73.3% of them suffered acne episodes in a moment of their life [95].

Acne in most of the patients is evaluated as a hormone-sensitive disorder [94]. The microbial colonisation, changing in the pH of the skin, sebum excess secretion, hyperkeratinisation, hormonal mechanisms, inflammation processes related with the action of the immune system, genetic influences and not least the mechanisms produced by diet-anxiety-stress triad are factors involved in acne development [96,97].

### 3.2. Current Therapeutic Models and Challenges

Acne can be presented as a pathology with superficial forms that can evolve through moderate or severe cases [98]. The skin aspect will be affected by the development of papules, comedones, pustules and their degenerated nodular and cystic forms, associated with erythema, cuperosis, inflammation of the lesions and scarring as a result of the mechanisms involved in acne pathogenesis [99]. In this direction, the current therapeutic outcomes are based on the selection of topical formulations associated or not with systemic therapy in severe cases. The main classes of anti-acne agents are antibiotics, antiseptics, retinoids and hormones. In uncomplicated forms, good results are gained with topical antimicrobials, while moderate cases need the addition of a topical antibiotic or retinoid. In severe and refractar acne types, the dermatologic algorithms are more complex, using topical antibacterial and systemic therapies with antibiotics or retinoids, associated with hormonal therapy as a last choice [93,98].

Furthermore, a disadvantage that can influence the adherence of a patient consists in the use of more than two pharmaceutical formulations to provide a synergism between APIs that act on different mechanisms of pathogenesis [100,101]. An improvement along this path consists in the development of some commercial products with combinations of APIs in low concentrations that can assure a better compliance [102]. On the other hand, the topical bioavailability of actives from conventional preparations is poorer and can be considered as a clue of therapy inefficacy, opening a way through the development of modern formulations based on vehicles that can reach the affected site by crossing the SC barrier [103,104]. In the management of acne treatment based on the introduction of modern pharmaceutical systems with a controlled release action, tretinoin was prepared in microspheres-based gel systems or as a micronized product, being available on the pharmaceutical market [104]. Consequently, based on the APIs usually used in acne therapy which are presented in Table 1, smart formulations could be developed with superior therapeutic outcomes.

It can be observed that current acne therapy approaches are based on the prescribing of classical formulations like topical solutions, creams, and gels. Among these therapeutic solutions, gels are the most appreciated in practice due to their superior bioavailability compared to creams, being characterized by a good tolerability and absorption [137]. Looking on the adherence in topical acne therapy, it can be observed in the same manner the influence of adverse symptoms. Regarding an actual study of Sevimli Dikicier on 250 subjects in a clinical hospital from Turkey, the discontinuation of acne treatment was analyzed using questionnaires with key factors that influence the patient response to therapy [138]. It was observed that 45.6% of the subjects have abandoned the therapy, signaling that side effects or absence of visible effects are the main reasons for neglect [138]. To conclude, adherence can reach a high level when skin response to therapy is positive and rapidly obtained.

Nevertheless, superior profiles and a reduction of topical adverse reactions can be acquired when the active substances will be prepared in smart vehicles with the aim to resolve the limitations of each compound. In addition, some studies have shown a transition from systemic therapies (that usually, beside the curative effects, can promote serious side effects) through topical therapies that can include the traditional systemic API, targeting it at skin site to ensure the expected healing effect, with minimal adverse reactions. Their integration at nanoscale domain using physical and chemical methods must be a helpful solution.

As a comparison with coarse dispersions that are used for a long time in clinical practice, nanosystem technology uses the accumulated knowledge and practical experience and transpose it by manipulation of matter at nano dimension. Hence, in accordance with the National Nanotechnology Initiative, this field of research encompasses the manipulation of matter with dimensions in the range 1–100 nm with the aim of achieving nanomaterials for diagnosis and treatment [139,140].

## 4. Microemulsions in a New Vision for an Optimized Acne Therapy

New modern formulations that contain actual anti-acne agents for potential dermatologic therapy represent better alternatives that must be studied for integration in clinical practice. Here can be distinguished a class of vesicular formulations which are defined as lipid-based nanosystems that include liposomes, transfersomes, niosomes, invasomes, ethosomes, cubosomes, sphingosomes, aquasomes, ufasomes or Leci Plex systems [141,142]. Besides these, superior effects were discovered by formulating systems like hydrogels which can incorporate nanoparticles [143].

Liposomal formulations are spherical vesicles with a double lipophilic membrane based on phospholipids and cholesterol. The lipidic envelope will surround a hydrophilic core, resulting in spherical vesicles [144]. For the rest of vesicular systems above mentioned, structural differences are specific considering the presence of non-ionic surfactants [145], cathionic surfactants [146], alcohols [147,148], terpenes [148] or amphiphilic lipids [149] beside phospholipids and/or cholesterol found in liposomes. The possibility to entrap hydrophilic or lipophilic substances is considered an advantage that led to multiple designs of anti-acne formulations. Favorable results considering API solubilization, protection and release were observed for liposomal formulations with tretinoin [150] or adapalene [151]. For salicylic acid [152] or rhodomyrtone [153] positive results were observed on bacterial inhibition. Other vesicular architectures were proposed as alternatives: transferosomes with clindamycin [154], niosomes with dapsone [145], invasomes with dapsone [148], ethosomes with azelaic acid [147], cubosomes with erythromycin [149] or LeciPlex with spironolactone [146].

Even if vesicular formulations are popular in this area of medical applications, several drawbacks may exist in their projection and let us to orient our interest on a different class of vehicles: a dimensional range of 50 nm until 500 nm, predisposing vesicles to hydrolytic and oxidative reactions; the presence of aggregative phenomena that can lead to unexpected dimensional changes; and the methods of preparation are laborious and expensive [155,156].

The scientific literature studied revealed multiple examples based on the formulation, preparation, and evaluation of microemulsions as ideal vehicles with multiple advantages for targeting anti-acne compounds, with positive results after the evaluation process.

With an abundant pattern of studies and multiple applications in pharmaceutical domain, microemulsions and nanoemulsions are modern colloidal dispersions with several advantages. The increase of bioavailability of active substances can be obtained in this way. Both systems have common characteristics with classical emulsions, but the addition of a co-solvent named also cosurfactant will generate unique vehicles that are capable of resolving the limitations of API integration in topical systems [157]. With referrals to particle dimension for micro- and nanoemulsions, particle dimension of 10–100 nm is specific for microemulsions, while in the case of nanoemuslions, particles dimension can reach 200 nm [157,158]. Other differences between them can be observed regarding the thermodynamic stability and the preparation methods. If microemulsions are thermodynamically and kinetically stable having the property of spontaneous generation, nanoemulsions are kinetically stable, but thermodynamically unstable systems. In the last case, the method of preparation requires the use of high-pressure homogenizers or proper sonication methods [159,160,161].

Considering these statements in the matter of nanosystem differentiation, it can be appreciated that microemulsions are fine systems with appropriate characteristics which will be further explained in what follows. Their introduction in dermatologic therapy can open new horizons toward specialized delivery, influencing in a positive manner the evolution of a skin condition like acne.

### 4.1. General Concepts

Microemulsions were empirically discovered in the past century, in 1943, when T. Hoar and J. H. Schulman have generated a monophasic and clear system using emulsion titration method with n-hexanol [162,163]. This was the first step for future development of smart colloidal systems named microemulsions from 1959 until today. Their initial utility at that moment was concentrated on the development of oil recovery techniques based on chemical approaches for industrial applications [164,165,166]. The evolution of the actual research methods sustains the knowledge and the discovery of unknown concepts which can help in the interpretation of physical phenomena, their physical structure and the mechanism of function for medical and pharmaceutical applications.

As a comparison with classical emulsions, microemulsions shall be differentiated, considering the following key properties: composition type, particle dimension, and thermodynamic stability. Emulsions are coarse dispersions formed by two immiscible phases which are stabilized using an emulsifier. The internal phase (discontinuous) is dispersed in the external (continuous) phase known also as a dispersion medium. The particle dimension can vary in the range 1–100 μm and is specific for opaque systems with a high interfacial energy. On the other hand, the interfacial tension has low values, assuring the stability of dispersed particles in the continuous phase [167]. The emulsion systems have a long tradition in topical application, used almost as fluid vehicles or as semi-solid forms in skin care or in dermatologic treatments [168]. A deficiency which can explain the short period of use for emulsions is the thermodynamic instability, consisting in phenomena like flocculation, coalescence, creaming, sedimentation, or Ostwald ripening. In addition, emulsion formulations are not proper for active substances with formulation challenges [167].

Microemulsions (MEs) are defined as microheterogeneous dispersions with a high thermodynamic stability. With a transparent appearance, the systems are observed as monophasic and isotropic structures basically formulated with an oil phase and an aqueous phase, stabilized by a mixture formed with a surfactant (S) and a cosurfactant (CoS) [165]. The clarity and homogeneity are physical markers for their dimension domain, placed in the range of 5–100 nm. The terminology of microemulsions type has a similarity with that of emulsions. Hence, the oil in water (O/W) MEs result when oil particles will be dispersed in an aqueous phase in the presence of a S/CoS mixture, while the water in oil (W/O) MEs will result in the dispersion process of water droplets in an oil phase, stabilized by a proper S/CoS mixture which will assure a superior stability [169,170]. The bicontinuous microemulsions are a particular type that requires equal amounts of oil and water in the system and are typically formed at the phase inversion temperature [171]. From a practical point of view, the transitions of ME from one type to another and their behavior are explained using the Winsor phase concept and pseudoternary phase diagrams. The study of the internal structure of microemulsions represents an important area in the research of ME thermodynamics [172,173,174].

The advantages of microemulsions are always analyzed at the beginning of a formulation process, being correlated with the actives that will be integrated and the resulting final product which can be defined as a simple system in composition, but complex in structure. In Table 2, are presented all the advantages that characterize the microemulsions as ideal vehicles for a topical delivery of active substances.

### 4.2. Physicochemical Concepts

One of the most important characteristics of microemulsions that provokes great interest in their study is the thermodynamic stability. In this direction, were formulated five theories that can guide the research through a deeper way of understanding the behavior of MEs. Here are distinguished the thermodynamic theory, the interfacial film theory, the theory of micellar state, the solubilization theory and the theory of bicontinuous microemulsions, from basic concepts through recent discoveries [182,183]. Each of these principles can offer preliminary answers about microemulsion structure and stability which can be continued by personal discoveries rallied to a specific group of systems.

The first theory, describes thermodynamic processes that occur in microemulsion systems, using the equation of free energy, as can be seen below [184]:

(1) Gf =γ·a−T·S

The energy of the system is dependent of the interfacial tension (*γ*) that usually has extremely low values, being exerted at the surface of the particles (*a*) immersed in the dispersion medium, and is characterized by temperature (*T*) and an increase in the entropy (*S*). As the surface of the particles become larger, the stability of the system will be increased [184,185]. In addition, the interfacial film generation theory sustains the null or even negative values of interfacial tension promoted by the addition of cosurfactant in the system [184]. In this way, in three steps can be explained in the formation of the interfacial monolayer as a stability promotor for microemulsions:

1. In a system composed of oil and water, the surfactant will promote a low interfacial tension at the interface between oil and water, resulting thus a monomolecular film [186].
2. The addition of a cosurfactant, will decrease the initial value of interfacial tension. The cosurfactant will be concentrated at the interface, among surfactant particles [186,187].
3. The system will be characterized by a free energy that will assure the microemulsifying process of droplets with a size of 1–100 nm which cannot be observed at a macroscale level, but only using fine experimental techniques like transmission electron microscopy (TEM) [185,188]. In this way, will be discovered particles with a specific assembly. As an example, in a study of Reis et al. were formulated O/W microemulsion systems that can load babassu oil 12.2% as an active oil component with anti-inflammatory effects. The analysed formulation was based on a mixture of two surfactants, Span 80 and Kolliphor EL, in combination with propylene glycol as a cosurfactant with a total amount S/CoS mix of 48.8%, in a water medium of 39%. Using TEM imaging, the internal structure was visualized, confirming the particle architecture, dimensional data and the type of MEs. Thus, were observed babassu oil phase droplets covered with the monolayer stratum of tensioactive mixture and embedded in the water medium [189]. Furthermore, O/W microemulsions for dermal and transdermal delivery with a 2% flavone extract of rhizoma arisaematis with analgesic properties were formulated using a vehicle composed of a S/CoS mixture with Cremophor EL 9–27% and Transcutol 8–27% with stabilizing properties for ethyl oleate particles 4–8% in a medium of 60% water. TEM analysis revealed the presence of spherical particles of oil phase with a dimension under 100 nm, stabilized in the aqueous medium [190]. In the same direction, confocal laser microscopy can be a useful tool with application in material structure analysis, being largely accessed for microemulsion characterization [191].

**Figure 5:**
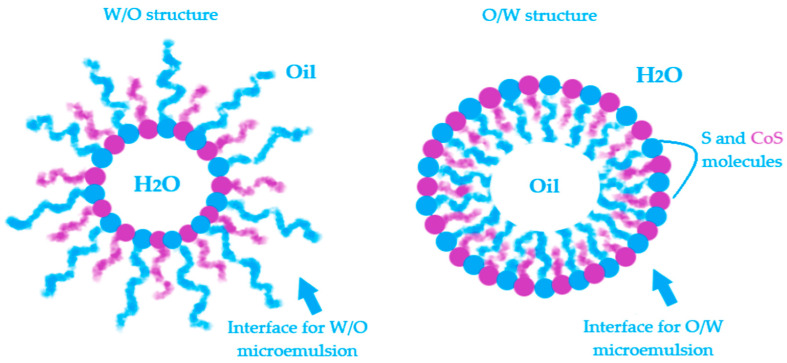
Exemplification of model structure with particles as a part of water in oil microemulsions and oil in water microemulsions and the specific orientation of surfactant and cosurfactant at the interface.

The interphases O/W and W/O are defined by a surface curvature of the monomolecular layer which is oriented as a function of oil and water content and the affinity of surfactant for hydrophilic and lipophilic groups [192,193]. In Figure 5 is exemplified a model for both W/O and O/W microemulsions and the placement of surfactant and cosurfactant at the interface.

The stability of microemulsions can be argued for in the same manner using the theory of elastic masses [193]. In this way, imagining microemulsion droplets as ideal spherical entities placed in a continuous phase, it can be presumed that two main characteristics are the elasticity and a proper rigidity against particle distortions [193]. The concept of elastic particles may give an input to the research of particle size using methods that can offer information about mean particle dimension, polydispersity index, interfacial tension or solubilization capacity [193,194].

Three elasticity constants are specific for microemulsion particles: firstly, the spontaneous curvature which influences the phase type and promotes stability. The solubilization capacity will be appropriate when the spontaneous curvature value will be smaller and is associated with many particles stabilized with a high amount of surfactant. Secondly, the rigidity constant, sustain the action of surfactant to resist to possible and undesirable curvature modifications. The rigidity constant increases when the third constant, the deformation (saddle-splay) constant, decreases to stabilize the system [193,195,196].

The theory of micellar state brings in front the idea of micelle generation as a common element between micelles and microemulsions. In both cases, it is selected a surface tension modulator (S). The addition of the CoS in microemulsions will make the difference among the two systems, obtaining complex structures [197]. An actual method to differentiate micellar structures and microemulsions it was proposed to be Taylor dispersion analysis which suppose the introduction of the samples in a capillary, followed by the measurement of hydrodynamic radius of the particles and their evolution in time [198]. In the case of some lipid microemulsions for oral delivery with Labrasol and Gellucire, the lipolysis process produced by pancreatic enzymes was studied in the same time with the dimensional evolution of particles, using Taylor dispersion analysis, offering clues in the matter of systems stability [199].

Apart from the classical composition of MEs, the solubilization theory suggests new insights in matter of ME spontaneous generation without surfactants, using the pre-Ouzo phenomenon [200,201]. A model system that can be easily exposed had three components: water, n-octanol as two immiscible substances one to another and ethanol as a co-solvent. Ethanol will be the hydrotropic co-solvent with solubilization power for the two immiscible compounds [200]. In a similar way were obtained ternary microemulsions based on water and eugenol in combination with ethanol [202]. As a co-solvent, ethanol will exert a hydrotropic action, transforming a turbid system into a clear one by a spontaneous emulsification as a rapid alternative to sonication methods [203].

The last theory of bicontinuous microemulsions brings to the fore the four Winsor phases of microemulsions [204]. Considering this statement, a microemulsion with a continuous hydrophilic phase can pass into a system with a continuous lipophilic domain when the oil phase will be added dropwise, experiencing an intermediate state of bicontinuity where may equally coexist hydrophilic and lipophilic domains [177]. The hydrophilic and lipophilic zones are chaotically interconnected and stabilized by surfactant. This ME was observed in a study of Kogan et al. for microemulsions formulated with triacetin, D α-tocopherol acetate, ethanol and Tween 60. The system was diluted with the aqueous phase, sustaining the transition from W/O type ME (known also as inversed phase ME) to O/W ME with the normal phase [205]. In this direction, the knowledge of Winsor phases serves as a guideline in the process of formulation and preparation of ME, helping to adjust the composition of the designed systems [206].

**Figure 6:**
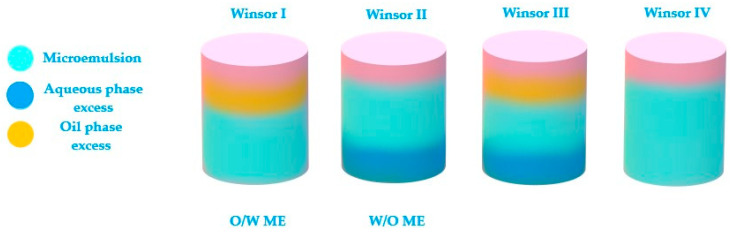
Schematic representation of Winsor phases I, II, III and IV, explaining the transition of phases from Winsor phase I to Winsor phase IV to obtain an ideal system.

According to Figure 6, the Winsor phases found in ME formulations can briefly be described. Winsor phase I systems are O/W microemulsions in an equilibrium with an excess of oil phase in the superior area of the vial. Winsor phase II systems are W/O microemulsions with an excess of water phase in the inferior area of the vial. Winsor phase III systems defines a ternary mixture at equilibrium composed of a microemulsion in the middle area, an excess of oil phase in the superior zone, followed by an excess of aqueous phase in the inferior area. Furthermore, the ideal Winsor IV system will contain a monophasic domain, without phase excess [174,207].

### 4.3. Formulation of Microemulsions

In the formulation process of microemulsions, the attention is focused on the mixture of S/CoS and the oil phase which will be associated with a proper amount of aqueous phase, commonly selected, the distilled water [165].

A surfactant, also named emulsifier, defines a molecule with specific properties of surface tension modulation, exerted in the system where will be integrated. Structurally, the molecule is composed of hydrophilic and hydrophobic moieties, viewed as two opposite poles that assure a preferential orientation at the contact with particles of the system, in ME case, the oil and water molecules [208,209]. With respect to interfacial film theory, the surfactant will be the key molecule in the dispersion process, offering a proper flexibility for particles in the continuous phase due to the generation of the interfacial monolayer [209,210].

Chemically, surfactants are classified as ionic (anionic, cationic and zwitterionic) or non-ionic species. The ionic surfactants, at the contact with the polar phase will generate a double electric layer, while non-ionic compounds will form dipole and hydrogen bonds [211,212]. It is important to mention that anionic and cationic surfactants are not recommended in cutaneous delivery systems due to their irritative potential. Thus, non-ionic surfactants are generally selected for topical products, having a good solubilization power for APIs and a reduced toxicity [211,213].

The hydrophilic–lipophilic balance (HLB) values calculated according with the Davies’ rule, as a function of head and tail groups of a surfactant, sustain its selection for a ME system. An HLB under 8 is attributed for surfactants used in W/O systems, while surfactants with HLB over 10 are suitable for O/W MEs. Stable systems are formed also by mixing two surfactants with different HLB values, as well as in emulsion formulation case [214]. According with the Bancroft rule, which is generally accepted in ME design too, the phase in which the surfactant is most soluble represents the continuous phase [215].

Specific for microemulsions is the high amount of S/CoS mixture selected up to 70%, differing from conventional emulsions where the emulsifier is integrated up to 10–20% [174,216]. An elaborate analysis is required in the formulation of topical systems; thus, the ingredients must be non-toxic, non-irritating and biocompatible, according with GRAS (Generally Regarded as Safe) concepts [217]. Recent approaches are focused on the use of natural surfactants as a green alternative to synthetic species, associated with algorithms in order to decrease the high concentration of S/CoS mixture, maintaining in the same manner a superior stability for the systems [217].

As examples of non-ionic surfactants, sorbitan esters like Span 20, Span 40, Span 60 Span 65, Span 80 and Span 85 are usually selected to promote W/O microemulsions due to their low HLB values between 1.8 for Span 85 and 8.6 for Span 20 [211,212,218]. On the other hand, polysorbates are chosen in the formulation of O/W microemulsions due to their HLB values over 10. Here are classified Tween 20, Tween 40, Tween 60, Tween 80, Tween 85 as combinations between partial esters of sorbitol and its mono-/dianhydrides, condensed with ethylene oxide groups [211,212,218]. Tween 80 is largely selected in microemulsion design due to its biocompatibility. Its association with alcohol based cosurfactants like ethanol or 1-butanol implies some attraction phenomena that will promote the solubilization of two immiscible phases. In the study of Prieto and Calvo [219], this mixture was suitable to stabilize n-hexane/water systems. The surface activity will be modified, being correlated with an increase in dielectric constant and ionization grade. The repulsion forces of Tween 80 at the interface n-hexane/water will be diminished with the addition of the alcohol. In order to choose which alcohol is preferred for ME generation, Traube’s rule can be followed which considers that in a homologous series of surfactants, the addition of a -CH2- group will decrease the molar concentration required to promote a reduction in the surface tension [212,219,220]. In this direction, it was proposed that the solubilization power will be proportional with the chain length of alcohol, being important to assure a balance between the chain length of S and the sum of oil and CoS chain lengths. For pharmaceutical applications, ethanol can be safely selected with special consideration on structural properties of each phase [219]. This statement was observed in the same manner in the study of Chai et al., which tested the power of solubilization for Tween 20, Tween 60 and Tween 80 on a ternary system like 1-butanol/dodecane/brine. Considering that Tween 60 had a high solubilization effect on the system, it was pointed out that the sum of 1-butanol/dodecane chain lengths will be proportional with the chain length of Tween 60 [221].

Other candidates usually found in ME preparation are Labrasol (polyethylene glycol (PEG) derivative of medium chain fatty acid triglycerides C8-C10 of capric and caprylic acids) or Cremophor derivates like polyoxyl 40 hydrogenated castor oil (Cremophor RH 40) and polyoxyethyleneglycerol triricinoleate 35 (Cremophor EL, Kolliphor EL) [14,222,223,224,225].

Non-ionic surfactants derived from natural sources are sucrose- and glucose esters which are considered ideal candidates for microemulsion generation, intensively studied as biocompatible tensioactives in drug delivery. Their surface activity and biodegradability are properties that recommend them in the formulation of topical systems [226]. As an example, using a mixture of Mazol 80 (association of ethoxylated mono- and diglycerides) and sucrose laurate as a biodegradable surfactant, in association with water and peppermint oil, Fanun has suggested a potential system for solubilizing active principles [227].

Another alternative to synthetic surfactants could be phosphatidylcholine-derived products like soy lecithin or egg yolk lecithin which have HLB values in the range 4–6, being suitable for W/O microemulsions [228]. The literature confirms the use of lecithin until 10% in ME formulations [229,230]. Nevertheless, it is recommended to design mixtures of lecithin with non-ionic surfactants with an HLB over 10 for O/W microemulsions. Surabhi et al. have formulated O/W anti-acne microemulsions with tretinoin, using a surfactant mixture of lecithin 1% and Tween 80 30% with ethanol 10% as CoS, associated with isopropyl myristate (IPM) as an oil phase in aqueous medium [231].

The second component of surface-active modulator mixture is the cosurfactant (CoS), also found as co-solvent, which has the property to decrease the interfacial tension, promoting a proper flexibility for the particles [232]. Cosurfactants (CoSs) are studied as penetration enhancers, being greatly appreciated in skin delivery. In addition, the association with surfactant molecules will assure a high solubility for both hydrophilic and hydrophobic substances [177]. CoSs used in ME preparation are preferred to be medium chain alcohols C2-C10 [233]. A high stability can be obtained using CoSs with short, medium and branched chains C3-C5 [234]. Here are distinguished the most selected CoSs in the formulation of MEs: ethanol, isopropyl alcohol, n-butanol, propylene glycol (PG), glycerin, n-pentanol, polyethylene glycol 400 (PEG 400), diethylene glycol monoethyleter (Transcutol P) [235]. Propylene glycol and Transcutol P are often chosen as CoSs due to their biocompatibility, along with their additional solubilization properties for APIs which can be observed over a screening process. In a comparative study of Abd Sisak et al., the efficiency of Transcutol P and PG in the generation of a large region of ME were demonstrated, compared with PEG 400 for systems prepared with Brij 97, oleic acid and water [236].

The oil phase is considered a continuous phase for W/O MEs or a dispersed phase that can incorporate hydrophobic actives in O/W ME type. The oil phase will be selected according with the solubility of the API to obtain its delivery in an encapsulated form [237]. Isopropyl myristate, ethyl oleate and oleic acid are synthetic oils usually preferred in ME formulation [238]. On the other hand, the use of vegetable oils became an interesting approach for ME preparation, mostly preferred by those containing fatty acids with medium chains and a low molecular weight. Additional effects of vegetable oils consist in hydration promotion, skin protection and rejuvenation. These approaches were appreciated and followed in a study of Hortolomei et al., which was based on the development of MEs with avocado oil, associated with a S/CoS mixture formed with sucrose laurate and Transcutol P. An increased tolerability of the formulated systems was suggested, emphasizing their potential for skin delivery [237,239].

Recent ME systems were formulated using grape seed oil in a short study of Scomoroscenco et al., using a S/CoS mixture composed of Tween 80, Plurol diisostearique CG and ethanol. The introduction of grape seed oil as a lipophilic phase was suitable to obtain cosmeceutical microemulsions [240].

Pascoa et al., have prepared microemulsions using *Pterodon emarginatus* oil. It was found that microemulsions containing 5–10% oil phase exerted an anti-inflammatory effect which was superior to the oil used as a single remedy [241].

For the antioxidant and hydrating properties at skin site, olive oil was included in O/W MEs designed by Chaiyana et al. The effect of different cosurfactants like propylene glycol, ethanol, isopropanol, and PEG-400 on the generation of a larger area of microemulsion was observed, with a notable impact on their effect at skin site. Two optimal MEs with the following formulation schemes S/CoS/Oil/Water (%) were considered, namely: Tween 85 64%/Propylene glycol 16%/Olive oil 10%/Water 10% and Tween 85 64%/Ethanol 16%/Olive oil 10%/Water 10%. Propylene glycol exhibited a good influence on hydration due to its humectant properties, being comparable with a hyaluronic acid preparation, while ethanol sustained the antioxidant activity of olive oil [242].

It can be appreciated that vegetable oils can be good candidates for synthetic oil phase replacing, due to their implications in skin moisturization, skin barrier rebalancing, UV protection, which are essential for a damaged skin. Argan oil, coconut oil, jojoba oil, oat oil, pomegranate oil, almond oil, rose hip oil are vegetable oils that can be recognized as potential active species in acne alleviation [243].

The second type of oil species that are frequently selected for ME preparation, with a high impact in skin delivery, are essential oils (EO). The essential oils are products resulting from the extraction process of different parts of aromatic plants. Their active compounds can exert biological effects in the human body, offering therapeutic actions [244,245]. A main class of organic compounds which are found in the composition of essential oils are terpenes that will act on SC destabilization due to their lipophilic properties. The terpene structure and physicochemical particularities of the drug are two criteria that must be taken under consideration in the preformulation step. To assure a good penetration in skin layers, non-polar terpenes with a high grade of unsaturation are preferred for lipophilic actives, while the species with hydroxylic moieties, characterized by a minimal degree of unsaturation can be selected for hydrophilic drugs [246]. In Table 3, are presented the advantages of essential oils and their contribution in the formulation of anti-acne microemulsions.

Considering the antimicrobial effects of essential oils for application in acne treatment as a part of a pharmaceutical product, the potential of the following oil models can be appreciated: *Acacia dealbata* essential oil, *Achillea millefolium* essential oil, *Boswellia carterii* essential oil, *Camellia sinensis* essential oil, *Citrus aurantifolia* essential oil, *Commiphora myrrha* essential oil, *Helichrysum italicum* essential oil, *Laurus nobilis* essential oil, *Lavandula angustifolia* essential oil, *Mentha piperita* essential oil, *Myrthus communis* essential oil, *Ocimum basilicum* essential oil, *Jasminum grandiflorum* essential oil, *Santalum album* essential oil, *Pogostemon patchouli* essential oil, *Rosmarinus officinalis* essential oil, *Salvia lavandulifolia* essential oil, *Thymus vulgaris* essential oil, *Vetiveria zizanioides* essential oil, *Viola odorata* essential oil [250,251,252,253].

To exemplify the impact of essential oils in microemulsion formulation, in the study of Lv et al., ME systems were prepared with the aim to assess a high permeation of quercetin at skin level using a group of essential oils with anti-inflammatory properties and comparing their efficacy. The power of solubilization was analysed as well using peppermint oil, clove oil or rosemary oil. Hence, essential oils have improved the photostability of quercetin compared with a simple aqueous solution. From a preparation point of view, the essential oil was considered as an oil phase being mixed with an amount of S/CoS mixture formed with Cremophor EL and propylene glycol 2:1 and finally titrated with water. Over evaluation, peppermint oil MEs had a larger area than MEs prepared using clove oil or rosemary oil. On the other hand, clove oil and rosemary oil offered a protective effect, assuring quercetin stability. Quercetin was degraded in a proportion of 67% in an aqueous solution compared with only 7% degraded in the microemulsions [249]. A study by Ma et al. offered a perspective concerning the antimicrobial effects of microemulsion systems enriched with essential oils or an essential oil compound and the influence of formulation factors on their activity. Thus, the systems were formulated with cinnamon bark oil, thyme oil or eugenol in a soybean oil medium, using Tween 80 and equal amounts of PG and water. The use of a microemulsion vehicle can increase the level of minimal inhibitory concentration of cinnamon bark oil from 313 ppm (the value obtained for the use of oil alone) to 625 ppm (for microemulsion) on cultures of *Listeria monocytogenes*. The study draws attention to the manner in which Tween 80 and soy bean oil inclusion will decrease the antimicrobial activity of the essential oils which can be attributed to some hydrophobic interactions between Tween 80 molecules and the lipophilic moieties of essential oils, suggesting the importance of concentration control in the formulation process [254]. A superior antimicrobial activity can be sustained with a proper amount of surfactant that can assure an increase in bacterial cell permeability for the primary API [255].

### 4.4. Methods for Microemulsion Preparation

Microemulsions are considered adaptive systems which can be prepared without high energy consumption in an economic manner. The preparation methods at room temperature are based on two types of titration method which can be applied and adapted as a function of the selected phases, their concentrations and the type of ME that is desirable to be formulated [177]. In practice, the microemulsification technique supposes the application of two methods: the phase titration method and phase inversion method using the oil or aqueous phase [184]. In the first case, a phase titration can be performed using for example the water phase drop by drop on a previously mixture prepared with the S/CoS mix and oil where the API can be dispersed. The oil titration method is realized in the same manner, without changing phase behaviour [256]. A phase inversion method is applied when the experimental plan is based on the development of reversed systems, being approachable for emulsions and nanoemulsions, too. To obtain a W/O microemulsion, an excess of the dispersed phase (the oil phase) will be titrated in a system containing water and the tensioactive mixture. Initially, a point specific to an O/W microemulsion can be depicted. As it will be added to the excess of the oil phase, the water phase will become the dispersed phase in a continuous oil phase, resulting the W/O type [177,257].

**Figure 7:**
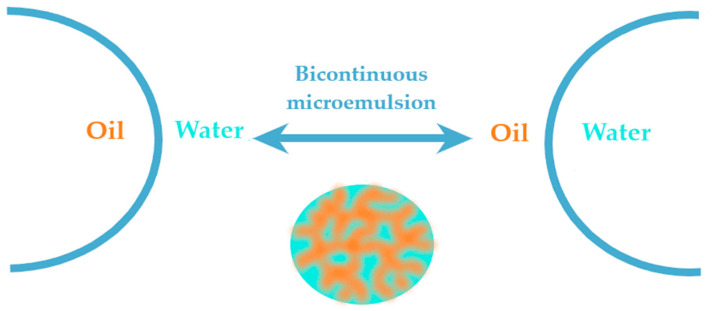
Schematic representation of phase transition in a microemulsion system with internal structure modification, from oil in water through water in oil type and vice versa, experiencing a particular bicontinuous state.

According with particle concepts previously exposed at the introductive theories section, the transition from O/W type to W/O type takes place with changes in curvature orientation, experiencing the particular state of bicontinuity, with the occurring of structural modifications as can be seen in Figure 7.

In the preformulation process, it is important to bear in mind the characteristics of the active substances, of the oil phase and S/CoS mixture and analyze their structure, to assess solubility data, according attention through the existence of possible interactions between molecules, along with pH or salinity influence, which may have further repercussions on the stability in time for the final product [258].

Considering the amounts selected for the aqueous phase, the oil phase, and the S/CoS mix, the region of microemulsion generation can be deduced using a pseudoternary phase diagram design. In laborious studies, when various proportions of tensioactives and oil phases are tested, the graphical analysis based on diagrams was found to be a key step to proceed an experimental design to obtain optimal microemulsions. In a large domain of studies, pseudoternary phase diagrams offer a good way for analyzing microemulsions stability, depicting zones which are specific to O/W, W/O and biocontinuous microemulsions [259]. To justify their stability, zeta potential evaluation is a required parameter, being defined as the potential difference between the mobile dispersion phase and the stationary stratum which is attached at the particle surface. As a function of ME composition, particularly the surfactant molecules, the high values negative or positive of zeta potential will be correlated with a high stability, while low values determine a destabilized system with a reduced half-life [260].

### 4.5. The Mechanism of Action for Microemulsions at Skin Level

Microemulsions are versatile systems that can promote a therapeutic effect when applied on the skin, being appreciated for dermal and transdermal drug delivery [175]. The formulation of the vehicle will influence the delivery of an anti-acne active at the affected zone, sustaining the activity not only in the outer site of the epidermis, but even in the deepest layers at dermis level, where structures commonly affected in acne flares are found, particularly the pilosebaceous unit and structural substances of the dermal tissue [261,262].

Considering the appropriate role of microemulsions for dermal delivery, these systems are known to be implicated in penetration activity, crossing the diffusional barrier of stratum corneum due to its main components, the S/CoS mixture along with the oil and the aqueous phase [263]. The oil in water type is almost preferred for anti-acne systems, due to its non-greasy structure, correlated with a low concentration of oil until 20%, which is the maximum required in dermatologic preparations. Here we can mention the study of Mortazavi et al., based on the preparation of microemulsions with tretinoin, where the amount of the oil phase was selected in proportion of 10% until 17% and evaluated considering its impact on particle dimension and skin delivery [264].

When a microemulsion is applied on skin, at the epidermal layer, the phenomenon of SC destabilization can be observed. The particles of the system have the capacity to intercalate among keratinocyte spaces. SC destabilization is promoted due to a high amount of tensioactive mixture which in addition will be implied in a decreasing process of interfacial tension at the skin surface. By diminishing the barrier function, accompanied by the creation of some passages of nanometric size, the passage of API particles can be promoted through the inside, with a diffusion process occurring [180,263]. Concerning the S/CoS mixture is important to assure an equilibrium between the maximum concentration selected in the system and implied in microemulsification, solubilization and diffusion process, obtaining at the same time a high level of skin tolerability [180].

A couple of practical examples can be rendered which can emphasize the action of ethanol, propylene glycol and Transcutol P on skin dynamics, considering some of the most selected excipients with penetration enhancement activity in microemulsion design. Using molecular models, for ethanol, the mechanisms of penetration enhancement were explained which can be the result of the interactions between alcohol molecules and the head groups of lipid species of stratum corneum, resulting hydrogen bonds. At skin site several phenomena supposed to be correlated with the mechanism of skin penetration are studied. Here we can mention: lipid extraction, alteration of protein domain, along with the increase of drug partition in skin lipids. Thus, in the experimental study based on the simulation of a bilayered membrane, ethanol was implicated in the extraction process of fatty acids and ceramides. As a function of its concentration, the membrane was gradually destabilized [265]. To continue, propylene glycol can be implicated in skin permeability modulation for both easy permeable and low permeable substances. The effects which belong to its destabilization properties on lipid organization are amplified in the case of PG association with hydrophilic compounds [266]. The association of PG with Tween 80 in different ratios will influence the passage of lipophilic compounds, being compatible with creating ME vehicles for drug delivery as was proposed in the study of Garcia Praça et al. The study was based on the solubilization in ME vehicle of vitamin A 0.05% and vitamin E 0.1% for skin target to obtain an anti-inflammatory effect [267]. In the same manner, it was observed SC dynamics and its reactivity to ME systems formulated with Tween 20 and Transcutol P which have stabilized an oleic acid/water system. Using infrared spectroscopy, it was proved that Tween 20 and Transcutol P influenced lipid extraction, SC destabilization and water upholding [268].

Transcutol P is an excipient that can assure the transition from a metastable system through a highly stabilized one. Transcutol is selected as a biocompatible co-solvent capable to induce structural modifications in colloidal systems. At the contact with SC, Transcutol will bind hydrophilic moieties of biological compounds present at this level. [269]. In the same manner it was reached a high interest in considering its solubilization properties, with positive effects on drug passage at skin site. As an example, Transcutol P was found to be a precious component that can promote monophasic systems even in O/W creams by decreasing the interfacial tension in the system from 26.73 mN/m through 3.42 mN/m, without the addition of an emulsifier [270]. More profound insights were discovered in a study by Björklund et al. on the effect of Transcutol P and dexpanthenol at SC level. Transcutol P can maintain a balanced hydration level in normal or dehydrated SC, with a similarity of action being found to the components of natural moisturizing factor (NMF) like urea or glycerol, along to its actions in the increase of ceramide head group mobility [271].

The use of natural surfactants combined or not with the non-ionic type and the use of vegetable oils can be an advantage to obtain biocompatible systems. As an example, a comparative study of Changez et al., based on the delivery of tetracaine in mice skin, proved that the addition of lecithin in ME systems can enhance the delivery at epidermal and dermal layers for tetracaine, compared with a topical solution with the same anesthetic [272]. Furthermore, the vegetable oils selected for ME systems can maintain an occlusive effect, with a similarity being discovered with mineral oils like paraffin oil. Changing the hydration gradient in the upper site of the epidermis by occlusive effect, can be a practical option for dermatologic preparations applied on dried skin [273]. *Camellia assamica* seed oil, enriched in fatty acids like cis-9-oleic acid, cis-9,12-linoleic acid, and palmitic acid was a good candidate for microemulsion preparation as an oil phase with moisturizing and antioxidant properties. Optimal microemulsions were obtained using Tween 85, in combination with ethanol (4:1) or propylene glycol (2:1), resulting O/W systems with 10% oil phase. It was proved that its incorporation in a ME vehicle can increase the biological effects at skin site, compared with the oil used alone [274].

In what follows will be presented a couple of interesting reports, based on the inclusion of anti-acne APIs in dermatologic therapy, as well as the advantages that may reside in their formulation and evaluation.

### 4.6. Application of Microemulsions for Anti-Acne Drug Delivery

Considering the numerous advantages exposed in the previous sections concerning the inclusion of MEs as ideal topical systems, several studies were based on the development of unique formulations that can be implied in a superior control of acne pathogenesis due to an optimal targeting of APIs at skin site, offering an alternative to conventional treatments [275]. Most of the projected systems were designed in order to resolve the solubility challenges of lipophilic actives like: vitamin E, retinoids, antibiotics and other antimicrobial agents like metronidazole or dapsone, offering in the same manner protection against undesirable internal processes by avoiding interactions and photochemical reactions. For hydrophilic substances, the easy passage through the stratum corneum can be handled for vitamin C, azelaic acid, nicotinamide, or hyaluronic acid, which are used as adjuvants in acne treatment.

Older evidence proved that microemulsions are superior to conventional pharmaceutical forms. Rangarajan et al. conducted a comparative study based on the delivery test of alpha-tocopherol 1% on a pig skin model. It was observed that the ME system delivered a higher concentration of the active than a hydroalcoholic gel, a gel, an emulsion or a solution [276].

Vitamin E, also found as alpha tocopherol, is considered a key element in skin nutrition, being largely used in dermatology for its antioxidant and chemoprotective properties. Its inclusion as an adjuvant in acne therapy can be justified by a supplementary action of vitamin E on keratinization mechanism [277]. Combined with vitamin C, a synergistic mixture [278] can be obtained that can act on the prevention of comedonal development, inhibiting *P. acnes* activity [277]. Vitamin C can form associations with vitamin E in biomembranes, protecting in this manner the lipophilic vitamin from oxidative stress [278]. A study by Rozman et al. was based on the development of microemulsions with both vitamin C and vitamin E, resulting three types of systems: O/W, W/O and gel-based systems. The stability was assessed in all the types, but was superior for the gel, due to the presence of the thickener that was implied in structural changes. From a rheological point of view, a transition of ME was observed from fluid one through a system characterized by thixotropy, influencing the quality profile, and improving dermal delivery [279,280].

Vitamin C can be used itself as an adjuvant for acne treatment. In topical delivery, beside the antioxidant activity, its implications in collagen synthesis, complete with anti-inflammatory properties will bring additional benefits to sustain the regeneration in acne affected skin, by regulating keratinocytes differentiation [281,282]. Numerous attempts were conducted with the aim to entrap vitamin C in systems like liposomes, nanoparticles, micelles or hydrogels for topical delivery [281], but microemulsions were found as essential systems that can protect and target vitamin C at skin site, increasing its antioxidant activity. According to the study of Pepe et al., vitamin C 0.2% and lycopene 0.04% were loaded in ME vehicles, formulated with decylglucoside as a biocompatible surfactant, combined with isopropyl myristate as an oil phase which was enriched with a second lipid fraction represented by monocaprylin, monolaurin or monoolein. It was observed that monocaprylin fraction has influenced the increase of antioxidant activity for vitamin C, compared with the last two lipid fractions [283]. A new study conducted by Ramli et al., concentrated on the development of another type of vitamin C based microemulsion with possible applications in inflammatory disorders like acne. Designed as a screening, the formulation process included the use of a group of non-ionic surfactants like Tween 20 and Tween 80, combined with PG or PEG 400 or glycerol as CoS, using D-limonene as an oil phase which can act also as a penetration enhancer, assuring a proper delivery of vitamin C in skin layers. The most convenient system was obtained using a mixture of Tween 20 and PG in a ratio 3:1, which can exert a large area of ME. The integration of vitamin C in the vehicle, as well as the water amount influenced the particle dimension [284].

Beginning with the group of retinoids, tretinoin is one of the most tested actives that can be incorporated in microemulsions. The study of Surabhi et al., previously pointed in the Section 4.3, offered a first perspective for the inclusion of tretinoin in ME vehicles. The systems were prepared with a mixture of two surfactants, lecithin and Tween 80, in combination with ethanol as CoS, using isopropyl myristate as an oil phase. The study was conducted assessing results from in vitro permeation and retention evaluation on laca mice skin, proving that the ME system exhibited the highest flux of 33.92 µg/cm^2^/h, compared with a MEgel, a classical gel, a commercial gel or a solution with the same active, as it can be seen in Table 4. On the other hand, the MEgel offered a maximum retention. The sustained release and the high value of retention parameter was correlated with the presence in the system of lecithin 1%. Considering its phospholipidic structure and the similarity with the skin components, lecithin may make a contribution to the increase of tretinoin retention in skin [231].

The study of Moghimipour et al. was concentrated on a physicochemical analysis of 8 MEs formulated using a mixture of 2 surfactants, Tween 80 and Labrasol, combined with PG in a ratio Km of 4:1 and 3:1. By contrast with the study before mentioned, in the oil phase represented by isopropyl myristate, was added Transcutol P as a co-solvent with the aim to solubilize tretinoin. The oil mixture had a maximum solubility of 4.85 ± 0.09 mg/mL. Using a 2^3^ full factorial design, it was discovered a ME model, observing that ME properties are influenced by the amount of each phase [285].

Similar data were depicted in the study of Mortazavi et al., previously referred to in Section 4.5, but with a high accent on a large group of surfactants and cosurfactants involved in a screening process, being defined in Table 4, along with important observations that have influenced the choice of optimal ME for tretinoin delivery. The formulation with Tween 80 33%/PG 12%/olive oil 15%/water 40% was found to be a suitable vehicle, with mean particle size of 319 ± 86.21 nm, compared with other formulations. The oil phase will influence in the same manner the final properties of the ME, where olive oil was found to be superior to castor oil or IPM [264].

As an application for the study of tretinoin delivery in skin layer, de Oliveira et al. developed an original liquid chromatography technique. The method was performed after a proper display of cream and gel pharmaceutical dosage forms on porcine skin membranes. The analysis of tretinoin in SC, viable epidermis and dermis was sustained using tape stripping and cutaneous retention methods. The highest level of release was obtained for gel formulation [286]. Based on this research, the utility of chromatographic methods can be appreciated in skin dynamics analysis and its transposition through ME systems.

A large perspective on ME formulation can be created considering the second retinoid for topical delivery, namely isotretinoin. A first study of Ramli et al. was based on the development of microemulsion vehicles, using a S/CoS mixture formed with Tween 80 and tetraglycol. The composition was enriched with bile salts, known as activators for an easy passage of the retinoid in skin layers [287]. Moreover, Patel et al. conducted a study centered on isotretinoin stabilization in ME systems that can assure its protection against photochemical reactions. From a kinetic point of view, isotretinoin degradation occurs rapidly in a methanolic solution medium, following a photochemical isomerization reaction. In order to obtain ME systems, mixtures of S/CoS were formulated as caprylocaproyl macrogol-8-glyceride/polyglyceryl oleate with Km 1:1, 2:1 and 3:1, which were associated with various ratios of IPM on the interval [0.5:9.5; 1:9; 1.5:8.5…8.5:1.5; 9:1; 9.5:0.5]. After the application of water titration method, points of stability were depicted on pseudoternary diagrams, being chosen an O/W ME model, where S/CoS mix had Km 3:1, as can be observed in Table 4 [288]. The study was upgraded with respect to patient adherence, particularly suggesting a way to develop ME systems with isotretinoin, considering parameters like enhancing drug solubility and tolerability with a superior target at the skin site. Positive resolutions are presented in Table 4, considering an O/W ME model formulated with Labrasol 24% and Plurol oleique 8% as S/CoS mix, combined with IPM 8% and water 60% [289]. Gürbüz et al. initiated a study to improve isotretinoin localization in skin as an alternative to systemic treatment by applying spectroscopic methods associated with confocal microscopy imaging [290]. A second study paved the way with the aim to obtain ME systems with superior physicochemical properties, using a pseudoternary phase diagram design. The study was projected as a screening, analyzing the impact of various CoSs on the formulation process, as can be observed in Table 4. Six groups of MEs were formulated using Labrasol as a surfactant, combined with Kolliphor EL or Kolliphor HS or Plurol oleique as a CoS. IPM was added, keeping the ratios of Oil:S/CoS in the interval [1:9; 2:8…8:2; 9:1] [291]. Novel approaches of Wani et al. showed the possibility to prepare O/W microemulsions with isotretinoin which can be administered by spraying. Thus, a ME-based spray can be a safe alternative for an easy application at skin site to avoid face-hand contact and to prevent microbial proliferation. The study initially started with a screening of various surfactants and cosurfactants in a ratio of 1:1, 2:1 and 3:1, where those preferred as a function of their solubilization power for isotretinoin are presented in Table 4. At the same time, basil oil can be a good solubilizer for API, being implicated in skin penetration enhancement and antimicrobial activity. The optimal system which was desirable to be administered was discovered applying a 2^3^ full factorial design, correlating S, CoS and oil amounts with particle size and drug diffusion. Beside the physicochemical analysis and in vitro release tests, the systems were tested microbiologically. Complementary to relevant experimental results a synergism was observed between API and basil oil, enriching the anti-acne effect. Once more, this study confirmed the quality of ME to protect isotretinoin against photodegradation, ameliorating its half line by 13 folds compared with a methanolic solution [292].

Recent attempts supported tazarotene delivery from ME systems. Patel et al. were concentrated on the impact of tazarotene MEs at skin site, choosing to conduct an optimization process for a group of systems. The optimal O/W system contained a tensioactive mixture of two surfactants: Labrasol, Cremophor RH 40 (1:1) 15%, combined with Capmul MCM 15% as CoS, isopropyl myristate 12% and water 58%. The system did not affect the skin structure and can be suitable for tazarotene skin target [293]. It is important to mention that Capmul MCM as an excipient with lipophilic character based on mono- and diglycerides with medium chain fatty acids, can have a solubilization function, being selected as a co-solvent for lipohilic drugs, but also as an oil phase in self-micro/nanoemulsifying systems [294,295]. The study was completed considering a proper delivery of the drug from a ME-based gel system. Beside the addition of Carbopol 971P NF as a gelling agent, the new system differed from the anterior case, due to a modification in the composition. Thus, isopropyl myristate 12% was replaced with Labrafac CC 10%. The water was adjusted at 60%. The permeation into the skin occurred in a controlled manner [296]. As an oil phase, Labrafac CC is suitable to be selected for O/W or W/O emulsions or microemulsions. It consists in a mixture of capric and caprylic triglycerides, appreciated for its emollient properties [297]. To continue, Nasr and Abdel-Hamid conducted an optimization study for ME with tazarotene, considering the impact of independent variables (the amount of S/CoS mix) over dependent variables expressed as particle dimension, rheological behaviour, with emphasis on the skin dynamics and API delivery. It was found that the ME vehicle which offered a high level for tazarotene deposition up to 75% was a W/O type with a S/CoS mix of 45%, oil 40% and water 15% [298].

Adapalene as a third-generation retinoid is highly appreciated for its action in acne affected skin, being at the same time a challenging substance that requires attention in matter of formulation, due to its poor solubility in water and photosensitivity. To access the follicular level, Bhatia et al. developed adapalene ME with a stability up to 1 year, using a mixture of S/CoS composed of Tween 20 and Transcutol, which will promote a fine dispersion of oleic acid particles in a water medium. Transfollicular target was proved using confocal laser scanning microscopy [299]. Two studies of Pajić et al. offered a large perspective in the field of adapalene formulation and evaluation for skin target using biocompatible MEs with natural-based surfactants. In the first study, from the group of sugar-based surfactants with non-ionic structure decyl glucoside and caprylyl/capryl glucoside (Plantacare 2000 UP and Plantacare 810 UP) were chosen and compared with the effectiveness of non-ionic ethoxylated species like Tween 80 (synthetic) and glycereth-7-caprylate/caprate, opening a niche area in the study of new natural tensioactives. Beside them, in the screening process, PG and Transcutol were selected as CoSs, along with C90 as an oil phase, named also Capryol 90, which contains propylene glycol mono/diesters of caprylic acid [300,301]. Applying the water titration method, 3 types of ME were formulated, as can be seen in Table 4, together with several data resulting from the characterization process, and it was concluded that alkyl polyglucosides are potential natural surfactants for ME design [300]. The second study was based on adapalene ME design using just an alkyl polyglucoside tensioactive, combined with PG as a CoS. Mainstay results were depicted over in vitro skin studies, concluding that ME with decyl glucoside can be a good solution for adapalene target in pilosebaceous follicle as a smart alternative to classical gels [302].

Antibiotics are largely prescribed in the treatment of acne in oral and topical dosage forms. Microbial resistance is found to be settled in prolonged treatment, being associated as well with skin reactions like erythema, scaling, dryness which will further influence the patient adherence. The efficacy of the proposed treatment may be compromised in the case of antibiotics like erythromycin or nadifloxacin [303,304]. For erythromycin, a study of Ochiuz and Hortolomei, revealed the importance of choosing the appropriate S/CoS mixture in the formulation study. ME systems were designed to assure a topical application of erythromycin, promoting a superior safety profile by avoiding the adverse effects of the API. The biocompatibility and the quality profile for MEs was assured by the selection of avocado oil as an oil phase which was a continuous phase for water particles dispersed under the action of a suitable tensioactive. In the screening, Tween 20 effects were compared with the properties of a defined amount of S/CoS mix based on Tween 20 and PEG 400, with the relevant results concerning their influence on ME properties being shown in Table 4 [305].

Following the same direction, Kumar et al. incorporated nadifloxacin in O/W ME using Tween 80 and ethanol as a S/CoS blend, in association with oleic acid as an oil phase. Even if nadifloxacin potency is reduced compared with clindamycin action on *P. acne*’s inhibition, incorporated in a ME, nadifloxacin can be targeted at the affected zone, at follicle level due to its components which promote the generation of nanosized particles in a domain between 95 nm and 560 nm [306]. Similar results were obtained in the study of Shinde et al., when nadifloxacin was loaded in a ME system composed of a mixture with a high solubilization power, namely Tween 80, associated with Transcutol P in ratios of 1:1, 2:1 and 3:1. The mixture was associated with various amounts of Capryol 90 as an oil phase on the interval of ratio [1:9; 2:8…8:2; 9:1]. Using a pseudoternary phase diagram design, an optimal ME was chosen for the integration of API. After the physicochemical analysis, the optimal ME system was prepared as a ME based gel, as can be seen in Table 4, using Carbopol ETD 2020 or xanthan gum. The integration of xanthan gum influenced the rheological properties of the optimized systems, obtaining an improvement in the spreadability and drug localization at skin site [307].

For azelaic acid delivery at the skin site in a proper formulation, with a superior effect than commercial gels and creams used actually, Peira et al. developed ME systems using the sodium salt of the acid which was implied in a higher flux and retention, compared with the acidic form [308]. New insights were emphasized in the study of Salimi et al., considering the impact of a ME vehicle for azelaic acid delivery, with respect to solubilization capacity, the impact of tensioactive mixture on ME generation, the assessing of a high release rate and the capacity of ME to promote skin tolerability, alleviating the side effects of the API. For the ME generation, a mixture of two surfactants, Tween 80 and Labrasol, was used combined with Capryol 90 as a CoS. Transcutol P enhanced the properties of the oleic acid, being implied in skin penetration and drug solubilization. A full factorial design was used to achieve information about the internal structure, and it was concluded that the composition will influence the ME properties, as can be observed in Table 4. Hence, by choosing a high amount of S/CoS mix, a large area of ME can be promoted, which can be easily depicted on the pseudoternary phase diagram [309].

Metronidazole as an antibacterial with antiprotozoal activity can be used successfully in the topical treatment of acne, particularly in the rosacea type which is manifested as a permanent vasomotor reaction, triggering vasodilation, inflammation, and telangiectasia [310]. A study of Tirnasksiz et al. developed W/O ME systems with metronidazole which exerted superior effects on rosacea symptoms than a classical cream. W/O MEs were prepared using a S/CoS mix composed of lecithin and butanol in a ratio Km 2:1, where lecithin, selected as a unique surfactant, is appreciated for its properties to promote W/O MEs. Beside them, isopropyl myristate and water were selected as opposite phases. Nine formulations were developed considering Oil:S/CoS ratio in the interval [9:1; 8:2…2:8; 1:9]. The transparent system was chosen which remained stable at the dilution test and it is presented in Table 4. The ME offered a therapeutic effect which was quantified and analysed on a randomized, vehicle controlled, double blind clinical trial on 12 patients. The reduction of erythema was observed in 50% of patients, while telangiectasia was cured in 38% of them, proving the clinical relevance of MEs formulated with metronidazole [311]. In other evidence, metronidazole emulgels were prepared starting with microemulsifyied matrices according to the study of Rao et al. and analysed using mathematical modelling. Important results concerning drug permeation and spreadability were dependent on the amount of each phase, the S/CoS mix formed with Acconon MC8-2 and PG with Km 2:1, using Capmul 908 as an oil phase. By applying the water titration method, were constructed diagrams considering the ratio of Oil:S/CoS in the same interval as the anterior case [9:1; 8:2…2:8; 1:9]. Xanthan gum was chosen as a gelling agent for metronidazole emulgels. It was concluded that, in accordance with Table 4, the amount of oil, S/CoS mix and the inclusion of xanthan gum will influence spreadability and the total release of metronidazole from emulgel systems [312]. In comparison with the previous study, propylene glycol can be considered a physiologically acceptable CoS that can replace 1-butanol in the formulation, increasing the biocompatibility of microemulsifyied emulgels.

Dapsone, a lipophilic compound with a powerful action on severe acne, was recently incorporated in ME-based gel vehicles, using thickening agents that can sustain a good sensorial profile by modifying the rheological properties. In the first study of Mahore et al., the ME vehicles presented in Table 4 were composed of a S/CoS mixture formed with Kolliphor EL and PEG 400, using Capryol and N-methyl-2-pyrolidone as an oil phase. Poloxamer 407 was added in the optimal system, assuring the formation of a viscous system and modulating the retention time at the place of application [313]. In a recent study of Beheshti-Mall et al., valuable results were obtained when dapsone was incorporated in an O/W vehicle formulated with Tween 80 as a S, a CoS formed with Transcutol and ethanol, using isopropyl myristate as an oil phase. By contrast with the anterior case, menthol was added as a penetration enhancer, while Carbomer 940 changed the rheological profile from liquid ME with Newtonian behaviour, through non-Newtonian ME which influenced the spreadability and skin dynamics. In accordance with the data presented in the Table 4, the addition of menthol 2.5–5% sustains the superior profile of the designed ME [314].

The study of Das et al. opened a way through ME design using tea tree oil or ethyl butanoate as solubilizing agents for ivermectin formulation. Systems were obtained with particles under 100 nm, influencing the target at skin site. Both oil species assured a proper stability for MEs, especially ethyl butanoate which positively increased ivermectin drug permeation [315].

A study of Boonme et al. was conducted in a comparative manner in order to achieve better results in the process of delivery of nicotinamide as a part of a W/O ME vehicle, compared with an isopropanolic solution. The system was prepared using Tween 80 and Span 80 in a ratio of 1:1, combined with isopropyl alcohol as a CoS. The S/CoS mixture promoted stabilization at the interface formed by isopropyl myristate and water. The ME was implied in the penetration phenomenon of nicotinamide, assuring proper skin retention in order to sustain its biological effect [316].

**Table 4:** Valuable results of experimental studies considering anti-acne microemulsion systems.

No.	API ME Type Method	Composition	Observations	Ref.
1.	Tretinoin O/W Water titration method	S1: Tween 80 30% S2: Lecithin 1% CoS: Ethanol 10% Oil: isopropyl myristate (IPM) 5% Water: PBS^1^ pH 5.5	Mean particle size: 110 nm. The release (µg/cm^2^/h): maximum for ME and MEgel compared with other formulations: ME > MEgel > gel > comm. gel > solution 33.92 > 31.54 > 28.67 > 24.28 > 22.33 The retention (g/cm^2^): greater for MEs, in the following order: MEgel > ME > Solution > comm. gel > gel 96.28 > 82.13 > 32.40 > 29.32 > 21.54.	[231]
2.	Tretinoin 0.05% O/W Water titration method	S1: Tween 80 S2: Labrasol CoS: PG Amount S1/S2CoS: 55–90% IPM + Transcutol P as oil phase: 5–30% Water: 5–15% Km 3:1 and 4:1 ME model: S/CoS 65%/Oil 30%/Water 5%	Mean particle size: 14–60 nm. The pH was situated in the near physiological range: 6.1–6.87. PDI^1^ values: 0.352–0.411, defining the homogeneity of the systems. Refractive index variation: 1.4397–1.4505, as a mark of isotropy. The release in 8 h from ME model was 49%.	[285]
3.	Tretinoin O/W Oil titration method	For screening: S: Tween 20, 40, 80, glyceryl stearate, stearyl alcohol, Span 20 or 80 CoS: PG, ethanol, isopropanol, PEG 4000 or PEG 6000 S/CoS amount: 45% Oil: olive oil, castor oil or IPM 10–17% Water: 38–45%	O/W MEs are suitable systems for tretinoin delivery. Span 20 and 80: not preferred for O/W systems. Glyceryl stearate and stearyl alcohol modified the viscosity of the systems. PEG 4000 and 6000 may increase the viscosity due to their high molecular weight. Ethanol and isopropanol may be reconsidered due to their fluidity promotion in ME systems. PG was preferred, along with Tween 80 and olive oil, promoting a maximum release of tretinoin of 82% in 24 h.	[264]
4.	Isotretinoin 0.5% O/W Water titration method	S: Caprylocaproyl macrogol-8-glyceride 31.5% CoS: Polyglyceryl oleate 10.5% Oil: IPM 4.0% Water: q.s. and optimal Km 3:1	Mean particle size (ME model): 45 ± 0.5 nm PDI^1^ value (ME model) was 0.145 ± 0.027, defining the homogeneity of the system. Refractive index (ME model): 1.329, as a mark of isotropy.	[288]
5.	Isotretinoin 0.05% O/W or W/O Water titration method	S: Labrasol 24–54% CoS: Plurol oleique 8–18% Oil: IPM 8–18% Water: 10–60%, and optimal Km 3:1 ME model had the following formula: S 24%/CoS 8%/Oil 8%/Water 60% Carbopol 971P NF 2% was added to create a MEgel	Mean particle size: 22.4 ± 0.2 nm (ME model)–85.9 ± 3.7 nm. pH was situated in the near physiological range: 6.30 ± 0.01–6.67 ± 0.06 (ME model). Conductivity values: 2.4 ± 0.1–68.6 ± 0.4 µS/cm, correlated with the amount of water and the type of ME. O/W type: attributed to the ME model. Viscosity for ME model increased from a fluid type (38 ± 0.07 cP) to a high viscosity (6.5 × 103 ± 2 × 103 cP), correlated with the addition of the gelling agent. Skin drug deposition was improved.	[289]
6.	Isotretinoin 0.05% O/W Water titration method	S: Labrasol 26.25–46.80% CoS: Kolliphor HS 7–12.5% or Kolliphor EL 7–12.5% or Plurol oleique 10–14.63% Oil: IPM 3.8–6.5% Water 34.94–61.14% and Km 3:1 and 4:1	Kolliphor EL and HS: promote a diminishing in particle dimension. API embedding did not affect ME’s properties. Conductivity values: 7.50 ± 0.06–77.65 ± 0.21 µS/cm, correlated with the amount of water and the type of ME. Viscosity: 22.15 ± 0.06–88.00 ± 0.19 cP. The Newtonian behaviour of MEs was correlated with MEs fluidity.	[291]
7.	Isotretinoin 0.05% W/O Water titration method	ME model formula: Kolliphor 22.5%/Ethanol 7.5%/Oil 8%/Water 61.95%/API 0.05%, Carbopol ETD 2020 0.75% and TEA ^1^ q.s. were added for ME gel	The ME model: used to prepare a ME based spray with isotretinoin and a ME based gel which. Particle size for MEspray: 68.79 nm The release (µg/cm^2^/h): maximum for MEspray: MEspray > MEgel > commercial gel 27.67 ± 0.12 > 21.81 ± 0.103 > 19.29 ± 0.34.	[292]
8.	Adapalene 0.1% O/W Water titration method	3 MEs types: 1. Plantacare 2000 15.9%/PG 30%/C90 15%/Water 39.1% 2. Plantacare 810 17.8%/PG 28%/C90 14%/Water 40.2% 3. Emanon 28%/Transcutol P 28%/C90 14%/Water 30%	Oil solubility of alkyl polyglucoside can be increased using CoS; Adapalene: good solubility in Plantacare tensioactives and Emanon EV and also in the oil. Adapalene embedding did not affect ME properties. pH values 7.38 ± 0.01–8.49 ± 0.01. Conductivity values: 61.6 ± 0.6 µS/cm (ME with Emanon) and 1022.0 ± 14.0 µS/cm (ME with Plantacare), correlated with the O/W type. Viscosity: 19.6 ± 0.27–27.90 ± 0.32 cP. The Newtonian behaviour of MEs was correlated with MEs fluidity. Skin application: favorable.	[300]
9.	Erythromycin 0.5% W/O Water titration method	S: Tween 20 CoS: PEG 400 Oil: avocado oil and IPM Water: 5–15% and Km 1:1	pH values: 5.02–5.35. Conductivity values: 11.98 ± 1.21–29.39 ± 1.92 µS/cm, correlated with the O/W type. Viscosity: 33.32 ± 1.58–58.50 ± 1.74 mPa·s. The Newtonian behaviour of MEs was correlated with MEs fluidity. PEG 400: implied in hydration and ME spreadability. The release: <40%/6 h (in vitro), influenced by the low water amount of 10%. PEG 400, an elevated water content and the adjusting of oil fraction should be reconsidered.	[305]
10.	Nadifloxacin O/W Water titration method	S: Tween 80 CoS: Transcutol P, with S/CoS 30–60% Oil: C90 10–20% and optimal Km 1:1 Were formulated 2 MEgels, using 1. xanthan gum or 2.Carbopol ETD 2020	Mean particle size: 65 ± 1.35–121.64 ± 1.35 nm. PDI^1^ values: 0.890 ± 0.124–1.132 ± 0.006, defining the homogeneity of the systems. S and CoS c% influence the particle dimension and drug entrapment. A high oil content will increase the particle diameter. Highest drug loading: for 10% oil and 60%S/CoS mix. S/CoS mix exerted penetration activity, altering the lipidic structure, assuring the passage of nadifloxacin.	[307]
11.	Azelaic acid O/W Water titration method	S: Tween 80 and Labrasol 60–70% CoS: C90 Oil: oleic acid and Transcutol P (10:1) 5–10% Water: 20–30% Transcutol P was added in the oil phase due to its solubilization power And Km: 6:1 and 4:1	Mean particle size: 48.05 ± 0.75–151 ± 1.1 nm. Viscosity: 72.5 ± 1.45–83 ± 1.7 cP. The Newtonian behaviour of MEs was correlated with MEs fluidity. Release: up to 42% in 24 h.	[309]
12.	Metronidazole 0.75% W/O Water titration method	Optimal ME: S: Lecithin 35.75% CoS: Butanol 17.86% Oil: IPM 26.86% Water: 18.86% and Km 2:1	Mean particle size: 11.6 nm. Conductivity: 1.5µS/cm, correlated with the W/O type. Viscosity: 457.3 mPa·s The Newtonian behaviour of MEs was correlated with MEs fluidity. Stability over 6 months.	[311]
13.	Metronidazole 1% O/W Water titration method	Optimal emulgel: Acconon 16.67% PG 8.33%, Capmul 10%, Preservatives 0.22%, Xanthan gum 1%, TEA ^1^ q.s, Water q.s. and Km 2:1	pH values: 6.0–6.9. Viscosity (optimal emulgel): 4568 ± 0.32 mPa·s (at 10 rpm) through 1087 ± 0.43 mPa·s (at 100 rpm). Rheological behaviour: expressed as shear thinning. The release (optimal emulgel): 93.16%.	[312]
14.	Dapsone O/W Water titration method	S: Kolliphor EL CoS: PEG 400, and S/CoS 20–43% Oil: Capryol and N-methyl-2-pyrolidone (1:1) 3–10% Water: 50–70% and Km 1:1, 2:1, 1:2 ME model had the following formula: S/CoS 32%/Oil 6%/Water 62% and Km 1:1	Mean particle size: 27.53 (ME model)–64.40 nm. pH: 5.6–6 (ME model); Conductivity: 13–15.8 µS/cm, correlated with the O/W type. Drug entrapment for ME model: 97.99 ± 0.040%. The release: 70 ± 0.09%.	[313]
15.	Dapsone 5.15% O/W Water titration method	For optimal ME: S: Tween 80 18% CoS: Transcutol P 20% and ethanol 18% Oil: IPM 4% Water: 34.85% Two ME models derived from the first optimal: by adding menthol 2.5–5% Carbomer 940 0.5% was added.	Mean particle size (ME without menthol): 48.3 nm; Menthol has increased the particle size due to its hydrophobicity, being localized at the interface. pH values: 5.46–5.50. Conductivity: 25.40–28.25 µS/cm, correlated with the O/W type. Permeation values for MEgel: 74.38 ± 0.70–106.25 ± 4.84 µg/cm^2^/10 h. Stability over 6 months.	[314]

^1^ Abbreviations: PBS—Phosphate buffer saline, PDI—polydispersity index, TEA—triethanolamine.

Concerning the use of essential oils in the development of microemulsions with anti-acne activity, Pansang et al. used the essential basil oil 3% extracted from *Ocimum basilicum* as an oil phase, associated with isopropyl myristate and integrated in a hydrophilic mixture, where the S/CoS mixture was Tween 80/PG in a proper amount of water. The ME was analyzed from a tolerability point of view on 30 patients, proving the safety profile of the vehicle at the skin site [317].

As a final remark in this direction, recent original research was conducted by Jantrawut et al., referring to the ME design using the antibacterial properties of orange oil. The MEs were prepared to be applied on skin as a part of a pectin film that can maintain an intimate contact with the tissue. The study revealed the importance of surfactant and cosurfactant screening in order to obtain large ME regions, proving that this may be one of the essences that form the basis of ME design. PG was found to be a suitable CoS that can be easily combined with Tween 80 in a ratio of 1:1 in order to promote a low interfacial tension, with a stabilizer effect amongst oil and water particles, and resulting particles in the nanodomain of 50–100 nm [318].

In the regeneration process of the acne-affected skin, the loss of structural substances like collagen or hyaluronic acid may be overcome using a targeted therapy. Microemulsions can be considered a modern alternative to invasive methods, based on the entrapping and transport of hydrophilic biomolecules with a low/high molecular weight in the skin layers by crossing the barrier of SC. A skin restructuring effect can be obtained using ME systems, after an appropriate selection of the formulation parameters [319].

## 5. Final Resolutions Considering Microemulsion Design

1. It can be appreciated that microemulsion properties are closely influenced by the formulation parameters, where each of the selected phases will sustain the final action of the API in the targeted zone.
2. Microemulsion systems will improve the localization of APIs in skin layers and can be optimized using Quality by Design principles.
3. The use of combined systems like microemulsion-based gels or emulgels and the use of natural derived excipients will improve the tolerability and the biocompatibility at the application zone.

Microemulsions are systems characterized by spontaneous generation, unconditioned by the input of a high energy in the manufacturing process, but the presence of tensioactive mixture which in some cases can reach a high concentration up to 70%, must be safe and tolerable, offering a biological support for drug target [320,321]. Considering the results of experimental studies, the increase in biocompatibility for topical microemulsions can be obtained following two objectives:

1. Modulation of the total concentration of tensioactive mixture, without modifications in the quality profile of the systems, using natural surfactants.
2. Integration of suitable substances that can enrich the quality profile of ME, based on their special properties, and here gelling agents, vegetable oils or biopolymers can be remembered.

As an example, Peng et al. marked the transition from a microemulsion system formed with an initial composition prepared with water, n-octane, n-octyl β-D-glucopyranoside and 1-octanol through biocompatible ME, where the last three compounds were replaced with biocompatible actives like isopropyl myristate, Plantacare 1200 UP and 1,2-octanediol, resulting in a formulation suitable for cutaneous delivery after the finding of a proper gelling agent that can promote an improvement in the rheological behavior [322].

It can be considered that in microemulsion preparation the perspectives are infinitely enlarged due to their versatility which can be observed as a positive characteristic, letting us discover and create simple systems for the target of dermatologic drugs. The transition from lab scale through a large manufacturing approach at industrial level requires the necessity to perform well-prepared work protocols, which must be based on a rigorous documentation that comprises inclusively the results from potential clinical trials. These statements can be considered future steps that will be analyzed and applied in order to bring microemulsion formulations to the front line of clinical practice.

## 6. Conclusions

To conclude, the study of nanocolloids offers a generous contribution to the evolution of topical treatments in order to sustain the healing process in dermatologic diseases. Microemulsions as a part of soft matter systems are characterized by high stability, biocompatibility, and tolerability, influencing in a positive manner the skin dynamics due to their special internal structure. Hence, the limitations of conventional systems can be overcome due to a balanced composition represented by the pseudoternary structure of oil-surfactant/cosurfactant-water. Multiple approaches were proposed to enhance skin bioavailability for both hydrophilic and lipophilic anti-acne compounds, offering a pathway through superior topical pharmaceutical options. By using the experimental design for optimization of the formulation process, along with the input of proper evaluation methods for each type of formulation, the complex pattern of microemulsion systems was proved to be an ideal vehicle for anti-acne drug targeting.

## Figures and Tables

**Table 1:** Active pharmaceutical ingredients (APIs) mostly used in acne therapy with specific mechanisms and effects and current pharmaceutical formulations used in dermatologic algorithms.

No.	API	Mechanisms and Effects	Formulations	Ref.
1.	Erythromycin -topical-	Antibacterial Bacterial resistance may occur	Solution with zinc acetate Gel with isotretinoin	[105,106,107,108]
2.	Zinc acetate -topical-	Anti-inflammatory Antioxidant	Solution with erythromycin	[109]
3.	Clindamycin phosphate -topical-	Antibacterial Bacterial resistance may occur	Gel, 1% Gel with tretinoin, 1.2%/0.025% Gel with nicotinamide 1%/4% Gel with benzoyl peroxide 1%/2.5%	[105] [110,111,112,113]
4.	Nicotinamide -topical-	Anti-inflammatory Moisturizer	Gel 4%/5% alone or with clindamycin	[111,112]
5.	Metronidazole -topical-	Antibacterial Anti-inflammatory	Cream, 1% Gel, 1%, 2%	[114,115]
6.	Ivermectin -topical-	Anti-parasitic Anti-inflammatory	Cream, 1%	[116]
7.	Doxycycline -systemic-	Antibacterial Bacterial resistance may occur	Capsules, 100 mg	[117]
8.	Minocycline -systemic- -topical-	Antibacterial Hepatic affection, vestibular events, autoimmune skin disorders may occur Induce photosensitivity	Tablets, 100 mg Foam, 4%	[117,118]
9.	Tetracycline -systemic- -topical-	Antibacterial Affected oral absorption when is administered with food Induce photosensitivity	Capsules, 250 mg Ointment, 3%	[119] [120,121]
10.	Cotrimoxazole -systemic-	Antibacterial Off-label use Just in refractar cases	Tablets, 400 mg/80 mg, 800 mg/160 mg	[122]
11.	Azelaic acid -topical-	Anti-inflammatory, antioxidant Increased erythema may occur Induce photosensitivity	Cream, 20% Gel, 15% Foam, 15%	[123]
12.	Benzoyl peroxide -topical-	Antibacterial Erythema, burning sensations, dryness may occur Induce photosensitivity	Gel, alone or with clindamycin	[113]
13.	Salicylic acid -topical-	Anti-inflammatory Keratoplastic Erythema, dryness may occur	Cosmeceuticals Associated with topical moisturizers Gel, 2%	[124,125,126]
14.	Isotretinoin -systemic- -topical-	Equilibration of desquamation process, by targeting retinoic acid receptors (RAR) (α, β and γ) Anti-inflammatory Erythema, dryness may occur	Capsules 10–40 mg Gel, 0.05% and 0.1% with erythromycin 4%	[93] [127,128]
15.	Tretinoin -topical-	Equilibration of desquamation process, by targeting retinoic acid receptors RAR (α, β and γ) Anti-inflammatory Erythema, dryness may occur	Cream Gel formulation with clindamycin Concentration domain 0.01–0.1%	[127,129]
16.	Adapalene -topical-	Equilibration of desquamation process, by targeting retinoic acid receptors RAR (α, β and γ) Anti-inflammatory Erythema, dryness may occur	Gel with benzoyl peroxide 0.1/2.5% and 0.3/2.5%	[127,129]
17.	Tazarotene -topical-	Equilibration of desquamation process, by targeting retinoic acid receptors RAR (α, β and γ), selectively on β and γ receptors Anti-inflammatory Erythema, dryness may occur	Cream Gel Foam, 0.1%	[127,130]
18.	Trifarotene -topical-	Equilibration of desquamation process, by targeting RAR γ Anti-inflammatory, comedolytic	Cream, 0.005%	[131]
19.	Cortexolone 17α-propionate -topical-	Antiandrogen Anti-inflammatory	Cream, 1%	[132]
20.	Spironolactone -systemic-	Antiandrogen Only women recommended	Tablets, 25–200 mg	[133]
21.	Cyproterone -systemic-	Antiandrogen Headache, edema, hepatic affection, menstrual cycle modification may occur Last line therapy	Tablets, 50–100 mg or combined with ethinylestradiol 2 mg/0.035 mg	[134]
22.	Dapsone -systemic- -topical-	Antibacterial High tolerability	Tablets, 300 mg Gel, 5%, 7.5%	[135,136]

**Table 2:** Advantages of microemulsion (ME) formulation for topical delivery of drugs.

No.	Advantages of Microemulsions	Ref.
1.	Thermodynamic stability, induced by the S/CoS mixture that provides a low interfacial tension due to a monolayer formed at the contact of oil particles with the water particles.	[175]
2.	Spontaneous formation due to an easy preparation method, without energy consumption; economic manufacturing.	[176,177]
3.	Incorporation of both hydrophilic and lipophilic compounds, resolving the solubility drawbacks for poor soluble drugs; MEs promotes their delivery at skin site in accord with a proper diffusion process.	[177,178]
4.	The solubilization capacity for API and the superior bioavailability are proportional with a high concentration of the S/CoS mixture introduced in the system.	[179]
5.	A high required concentration of surfactant, in association with a cosurfactant assure an easy passage through stratum corneum, acting as penetration enhancers; the barrier effect will be diminished.	[177,180]
6.	The selected API in microemulsion formulation is protected against hydrolytic and oxidative processes, being incorporated as encapsulated particles in oil or water fine droplets; MEs have a superior stability with improvement in half life.	[177,179]
7.	Incorporating APIs that are usually formulated for oral dosage forms and can be adapted in skin target. In this manner, it can be avoided the first pass liver effect and serious adverse reactions, promoting a localized action, without systemic effects.	[181]

**Table 3:** Advantages of essential oils (EOs) and their impact on anti-acne microemulsion formulation.

No.	Advantages	Ref.
1	EOs can be selected as an oil phase in ME or combined with a second vegetable or synthetic oil.	[247]
2	EOs can be a good alternative to chemical solvents.	[248]
3	EOs act as penetration enhancers at skin site, increasing the localization of drugs.	[246]
4	EOs have low toxicity, being GRAS recognized.	[246]
5	EOs protect the API from degradation reactions, improving its stability; the microemulsification will protect EOs from degradation and volatilization.	[248,249]
6	EOs can exert self-anti-acne action on microbial species like *P. acnes* or *S. aureus*.	[250]
